# Guayule (*Parthenium argentatum* A. Gray), a Renewable Resource for Natural Polyisoprene and Resin: Composition, Processes and Applications

**DOI:** 10.3390/molecules26030664

**Published:** 2021-01-27

**Authors:** Amandine Rousset, Ali Amor, Teerasak Punvichai, Sandrine Perino, Serge Palu, Michel Dorget, Daniel Pioch, Farid Chemat

**Affiliations:** 1 GuaTecs, 28 rue Xavier Bichat, 72000 Le Mans, France; amandine.rousset3@alumni.univ-avignon.fr (A.R.); mdorget@guatecs.com (M.D.); 2 Avignon University, INRAE, UMR408, GREEN Extraction Team, 84000 Avignon, France; sandrine.perino@univ-avignon.fr; 3 CTTM, Centre de Transfert de Technologie, 72000 Le Mans, France; ali_amor44@yahoo.fr; 4 UR BioWooEB-Biorefinery Team, CIRAD, 34398 Montpellier, France; sergepalu@orange.fr; 5 Faculty of Science and Industrial Technology, Prince of Songkla University, Surat Thani Campus 84000, Thailand; teerasak.punvichai@yahoo.com

**Keywords:** guayule, polyisoprene, rubber, latex, green extraction, green reagents, green chemistry

## Abstract

Natural rubber is an essential material, especially for plane and truck tyres but also for medical gloves. Asia ranks first in the production of natural rubber, of which the *Hevea* tree is currently the sole source. However, it is anticipated that this source alone will not be able to fulfill the growing demand. Guayule, a shrub native to northern Mexico and southern United States, may also contribute. This plant not only contains polyisoprene, but also resin, a mixture of lipids and terpenoids. This review summarizes various aspects of this plant, from the usage history, botanical description, geographical distribution and cultivation practices, down to polyisoprene and resin biosynthesis including their distribution within the plant and molecular composition. Finally, the main processes yielding dry rubber or latex are depicted, as well as the properties of the various extracts along with economic considerations. The aim is to provide a wide picture of current knowledge available about this promising crop, a good feedstock candidate for a multiple-product biorefinery.

## 1. Introduction

Plant chemistry has been receiving increased interest during the last decades, now becoming fully integrated to our economy. Indeed, the petrochemicals found today in most manufactured products, are being gradually replaced by products and materials derived from renewable resources. Elastomers are no exception to this trend, particularly rubber, an essential material of the 21st century [1].

Polyisoprene (PI, the polymer taken as a whole whatever its stereoisomeric structure and origin) can be used in two different forms, either as latex (the polymer being dispersed in water, as a white liquid) or dry rubber (obtained by latex coagulation) [in this review, “polyisoprene” applies to the polymer molecule, while “rubber” is used when talking about the material which is a mixture of polyisoprene and of other components like proteins and small molecules]. PI is used in more than 40,000 products, like tyres, medical gloves or condoms. Synthetic routes provide a large amount of PI and of similar polymers, but natural rubber and latex from *Hevea* (NR and NL, respectively) remain irreplaceable for some applications, like plane and truck tyres or medical gloves thanks to the peculiar *cis* chemical structure of the contained PI and to the presence of other molecules [2]. Indeed NR shows better dynamic properties, especially resilience (defined as the ability to undergo big deformations without breaking and to recover its initial form when the constraint is released), as well as very good resistance to abrasion, shock and tearing [3,4].

More than 2000 plants produce PI [5]. The best-known plants for their potential commercial interest are part of the families of *Euphorbiaceae* (*Hevea, Bentamia, Manihot*), *Asteraceae* (*Parthenium argentatum, Taraxacum kok-saghyz*) and *Sapotaceae* (*Gutta percha, Argania spinosa*) [6]. The more promising ones are Kazakh dandelion (*Taraxacum kok-saghyz)* and guayule (*Parthenium argentatum* A. Gray) [7,8]. This review will focus on guayule. As of today, a lot of scientific papers have been published [9], on different aspects of the plant, ranging from agronomy to extraction process, focusing on PI or even resin, a mixture of compounds also found in this plant.

**Figure 1:**
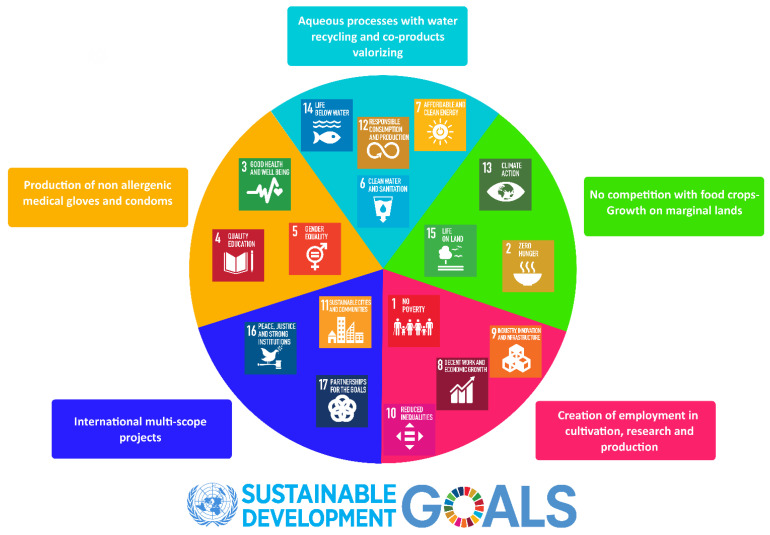
United Nations SDGs applied to guayule and their impacts.

Guayule is therefore an interesting species and it falls within the framework of the 17 UN Sustainable Development Goals (SDGs) (Figure 1). These SDGs were adopted by all United Nations Member States in 2015 and represent shared guidelines “for peace and prosperity for people and the planet, now and into the future” [10]. Indeed, guayule can be grown on marginal land, and its PI can be extracted under latex form by a water-based process to manufacture non-allergenic medical gloves and condoms, thus contributing to people’s health and safety; in addition, a range of co-products can be valorized [11,12]. The current interest on guayule leads to large international multi-scope projects, in view of setting-up a new production chain, potentially opening new jobs in agriculture, academia and industry.

## 2. History of Guayule

**Figure 2:**
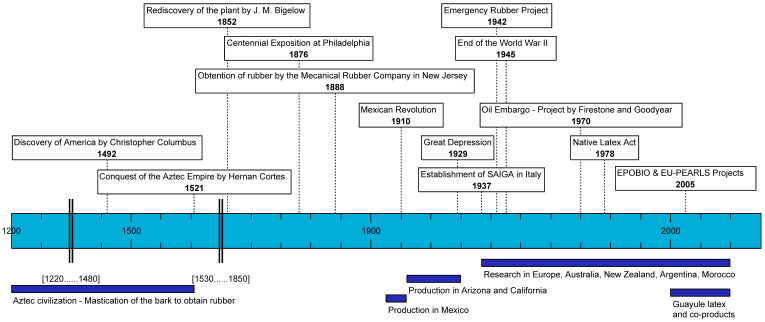
Guayule history timeline.

Guayule, also called “yerba de hule” is native to the desert of Chihuaha, located in Mexico and southern Texas in the United States. The name comes from the Nahuatl language (Aztec): *quahu* (wood, tree, forest) and *olli* (rubber). During the pre-Columbian times, it was basic knowledge that guayule contained an elastomer. The Aztecs used it to make rubber balls by mastication of the bark [13,14]. According to Lloyd [13], guayule was first documented by Bigelow, a member of the Mexican Boundary Survey party, in 1852. He submitted specimens he had collected in Texas to Harvard University, where Asa Gray named it botanically [15]. It was first presented to the public at the Centennial Exposition of 1876 in Philadelphia by the Mexican Government. On the same year, the Natural History Society of Mexico investigated the plant and reported the presence of good quality rubber [15] (Figure 2).

The first reported use of guayule, other than by the Aztec natives, was by the Mechanical Rubber Company, obtaining a “large quantity” of rubber, in 1888 in New Jersey. In the early 1900s, the first guayule factories were built in Mexico and rubber exported to the US. In 1902, the activities at the experimental laboratory at San Luis Potosi led to the establishment of a factory in Jimulco, representing the Compañía Explotadora de Caucho Mexicano. Guayule rubber (GNR) was put on the market, for the first time, in 1905. From that time on, extraction factories of various sizes were established in San Luis Potosi, Saltillo, Monterey, Gomez Palacio, as well as in Torreon and Jimulco [13]. Threatened by the Mexican Revolution, crops were transferred to California under the name of the American Rubber Producers, an incorporation of the Intercontinental Rubber Company. At that time, 50% of U.S. rubber were extracted from wild shrubs [16]. Wild harvesting led to gradual guayule depletion [17]. It was therefore decided not to use wild shrubs, but rather to establish fields throughout California and Arizona. In the 1920s, the Intercontinental Rubber Company in California produced 1400 tons of rubber. Production decreased with the Great Depression in 1929 [14,15]. On the other side of the Atlantic Ocean, new interest in guayule bloomed in Italy. The Italian Government signed a pact with the US Intercontinental Rubber Company, to establish guayule in Italy. Different areas in southern Italy and northern Africa were evaluated to assess a potential production. 5000 ha were identified as sufficient to fulfill the Italian needs. In 1937, the Agro-industrial Society of Anonymous Rubber (SAIGA) was officially established to develop guayule cultivation in Italy [18,19]. In 1942, *Hevea* rubber supply to the US from South-East Asia was cut off because of the invasion by the Japanese during World War II. The U.S. Government purchased the experimental records, seed stocks, and holdings of the Intercontinental Rubber Co, and the US Department of Agriculture established the Emergency Rubber Project: the plantation of 12,140 ha of guayule was launched and an extensive research program was initiated [15]. It stopped with the end of the World War II, since it was cheaper to import *Hevea* rubber from Asia with a better quality than to use local guayule rubber [15]. The 77th Congress ordered the destruction of the fields [16].

In Italy, the Italian Government decided to establish another society to study synthetic rubber (Synthetic Rubber Industry Limited Company: SAIGS, which in 1939 also incorporated the SAIGA. At that time, the production in SAIGA was only 1500 t/year and the synthetic rubber had increased drastically its share. Later the whole Italian rubber industry was moved to Germany. Thus, in 1950, the guayule development project in Italy was officially abandoned [18]. The focus on guayule rubber came back in 1970, with *Hevea* rubber prices rose due to the first oil embargo. Two large US tyre producers, Firestone and Goodyear, showed an interest in developing guayule rubber in Texas and Arizona. In 1979, the Guayule Rubber Society, was incorporated as a Texas nonprofit organization to foster and promote the production of natural rubber from the guayule shrub. It was reincorporated as the Association for the Advancement of Industrial Crops (AAIC) in December 1988 [18].

Mexico never stopped its research on guayule in Saltillo and, in 1976 new experimental tyres were manufactured. In 1977, the US National Academy of Sciences published a report showing the importance of this crop. One year later, it was followed by The Native Latex Act, “a bill to amend the Public Works and Economic Development Act of 1965 to authorize a program of research, development, and demonstration of guayule rubber (GNR) production and manufacture as an economic development opportunity for the southwestern States” [16,20].

In the 1990s, the interest shifted from crude GNR to latex (GNRL). Until then, the focus was on dry rubber obtained by a solvent extraction process, for tyres production. With the development of an extraction process in latex form at the US Department of Agriculture-Agricultural Research Service (USDA-ARS) [21], a water-based emulsion was obtained and new applications became possible: dipped latex gloves or condoms. The major interest of GNLR is its low protein content and therefore its non-allergenic or hypoallergenic properties [4]. In 1999, Yulex company was created, focusing on guayule latex. In 2008, the U.S. Food and Drug Administration (FDA) approved the use of GNRL for the manufacture non-allergenic gloves. At the same time, in Europe, the Realizing the Economic Potential of Renewable Resources-Bioproducts from Non-food Crops (EPOBIO) project (2005–2007), aimed to “design new generations of bio-based products derived from plant raw materials” [22].

The Production and Exploitation of Alternative Rubber and Latex Sources (EU-PEARLS) project, a continuation of the EPOBIO project, was launched in 2008 to study the development, exploitation and sustainable use of guayule and Kazakh dandelion, aiming at investigating the cultivation of the two crops in four countries: The Netherlands, Germany, France and Spain. Furthermore, in 2011, the first European study on guayule entitled “Agronomic evaluation of guayule cultivation in two northern Mediterranean areas” was presented at the AAIC meeting [18]. The EU-PEARLS project ended in 2012 with car tyres produced by Apollo Vredestein (The Netherlands), and medical gloves produced by CIRAD and its subcontractor CTTM (France) [23]. In 2013, Yulex entered a joint venture with Versalis and Pirelli in Europe, for the construction of an industrial plant in the southern EU, including testing GNR for tyres. Unfortunately, that plant was never built for economical reasons [19]. In the same year, Bridgestone announced its interest in GNR and its co-products. In 2015, first tyres made entirely of GNR were produced by Bridgestone [24]. At the same time, Cooper Tires and Panaridus developed plantations in Arizona and tyres were produced [25]. Today, several universities and research centers (USDA/ARS, University of Arizona and Ohio State University in the U.S., CIRAD in France, Keygene and Wageningen University in The Netherlands, University of Bologna in Italy etc..) and companies such as Bridgestone, Cooper Tires, Energyene, GuaySS, and the new start-up, GuaTecs, are working on guayule, in domains ranging from genetics and agronomy to the production of rubber and latex (Figure 2).

## 3. Botanical Description, Geographical Distribution and Cultivation Practices

### 3.1. Botanical Description

Guayule, *Parthenium argentatum* A. Gray [26], is a semi-arid perennial shrub, native to the Chihuahan desert in Mexico, that reaches a height of 0.3–0.9 m in the wild [27]. The genus *Parthenium*, member of the Asteraceae family (Table 1), encompasses 17 species with gray-green and silvery sheen leaves, but guayule is the only one that produces a significant quantity of natural PI [28].

**Figure 3:**
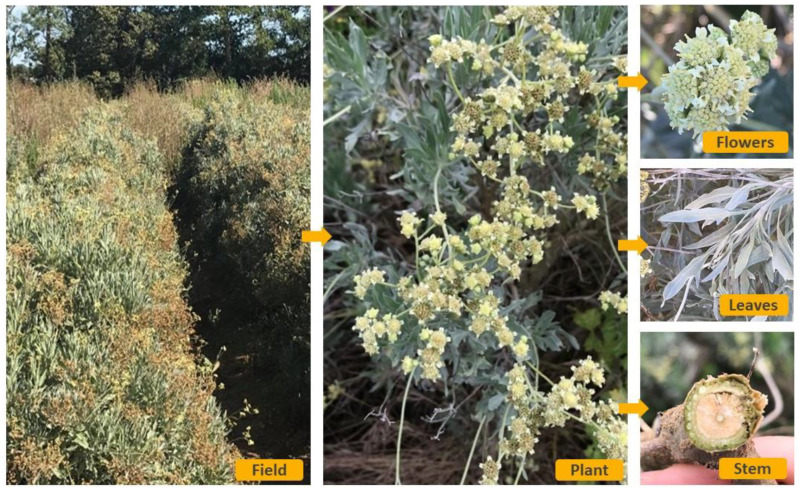
Field and various parts of guayule in Lansargues, south of France 2020.

Guayule flowers are arranged in heads or capitula. These heads are about 5 mm in diameter and contains five rays, producing one seed each, and a flower disk, producing pollen. When the seed germinates, we can see first a short primary stem, terminating in a long slender taproot. The cotyledons (first leaves) are circular and few millimeters. The primary stem grows and becomes dark red if exposed to sunlight, or green if not. The first leaves are closely crowded, ovate and green-gray, satiny sheen. In the field, they can reach a length of 7–8 cm and a width of 1.5 cm. The first inflorescence may occur during the first 6 months. The root develops in a strong tap root system extending in many strong lateral roots that can reach 1 to 2 m. From these lateral roots, “adventitious shoots”, called “retoños”, can expand especially on the thin-soiled, rocky slopes where the seedling growth is difficult [13] (Figure 3).

Guayule has much inherent genetic variability: plants with chromosomes numbers from 2n = 36 to 100 are known [16]. The guayule types 2n = 36 reproduce sexually with pollination [16]. It can be both wind and insect pollinated [15]. The others, “apomicts”, reproduce without requiring double fertilization: the embryo of the seed arises from a non-fertilized nucleus and grows genetically identical to the parent. Hybrids with useful characteristics can be developed [16].

### 3.2. Geographical Distribution

Native plants can live up to 30–40 years or more [16]. They can be found in the Mexican states of Zacatecas, Coahuila, Chihuahua, San Luis Potosí, Nuevo León, Durango and in the US states of New Mexico and Texas, in the areas next to the Big Bend [11,15,16]. The altitude of these areas varies from 610 m to 3500 m above sea-level. The plant tolerates little rain: an annual rainfall of 250 mm to 380 mm is enough. It resists to temperature higher than 40 °C and can withstand temperatures below −15 °C [3,13]. 

### 3.3. Cultivation

Guayule cultivation practices have been reviewed by Whitworth and Whitehead [14]. Guayule is adapted to hot desert environments and to sites with different soils (shallow, stony, calcareous and friable) and with relatively low concentrations of nutrients, but it grows best in well-drained soils and cannot tolerate waterlogging [16]. Some studies about the impact of irrigation have shown that rubber content decreases with irrigation amount, except with subsurface drip irrigation, where rubber yields are increased with irrigation amount, because the biomass increases too [29]. In the U.S, California, Arizona, New Mexico and Texas are suitable growing areas. Countries near the Mediterranean Sea, like France, Spain, Italy, Greece, Turkey, Israel and Morocco are suitable too [30]. Several other countries have also conducted guayule crop trials such as Argentina [31], Australia [32] and South Africa [33].

The first step of cultivation consists of collecting the seeds. They can be removed from the plant easily by hand or by mechanical means. Several vacuum harvesters have been developed, from the Intercontinental Rubber Company’s, to the Bridgestone/Firestone’s one, powered by an electric generator, mounted on a high-profile tractor. Once collected, it is important to break seed dormancy. An example of protocol to promote germination under acute osmotic stress, with a conditioning process follows. Seeds are left imbibing under aerobic conditions in a medium containing: 25% polyethylene glycol (MW 8000), 10 mmol gibberellic acid, 0.05% potassium nitrate, and 0.1% Thiram adjusted to pH 8.0 with a saturated solution of calcium hydroxide. They are then treated at 25 °C in continuous light for three to four days and air dried [14].

Seedling transplants are then produced in greenhouses. They are transferred in fields using typical transplanting systems. Direct seedling, cheaper, has also been successful. Mechanized techniques have been adapted for all aspects of guayule cultivation and Ray proposed to reduce the cost by clipping the plant instead of digging hole [34].

## 4. Distribution and Biosynthesis of Polyisoprene and Resin

### 4.1. Distribution of Polyisoprene and Resin in the Shrub

PI and/or resin are found in different sections of the stems, branches, roots, leaves and flowers. On average, there are 8% of P and 10% of resin in the plant (dry weight of wild shrub, older than 24 months). For Lloyd and Jasso de Rodriguez, two thirds of the PI are in the stems and branches, and one third in the roots. The leaves contain little or none PI [13,35,36]. Stem has 6.1% resin followed by roots (5.9%), branches (5.0%), leaves (3.0%), and flowers (3.1%) (dry weight of each part) [37].

**Figure 4:**
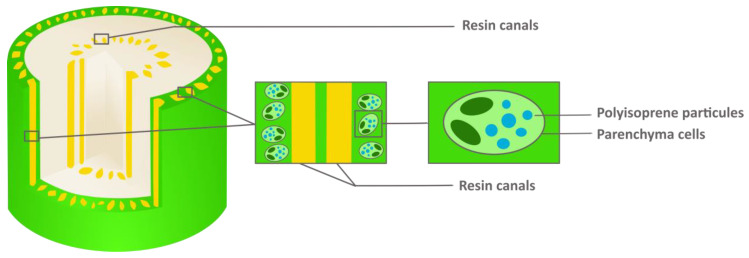
Distribution of polyisoprene and resin in guayule stem.

**Figure 5:**
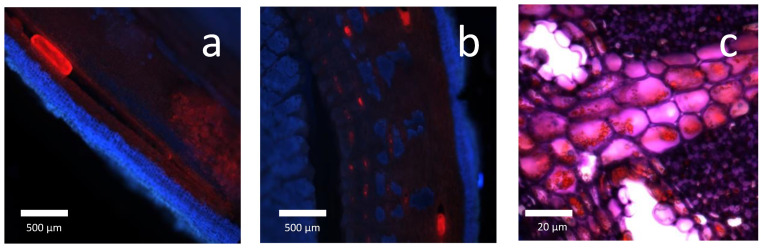
(**a**). Longitudinal section of a guayule branch (resin in red inside a canal, bark in blue)/(**b**). Cross section of guayule bark (resin in red, bark in blue)/(**c**). Particles of polyisoprene in parenchyma cells (polyisoprene in red) [36].

NR is contained in thin-walled cells, the parenchyma cells, located in the bark and the pith [3]. (Figure 4) It is suspended in the cell sap in the form of an emulsion, and this emulsion rapidly degrades upon contact with air [16]. Resin is accumulated in the resin canals in parenchyma tissue and in pith [38]. (Figure 5 [39]) For Hammond, it is principally in the bark of stems and roots, and only a little part can be found in the leaves [15]. Upon wounding, it exudes as pale-yellow tears [13]. The number of resin canals and epithelial cells increases yearly and thus the amount of PI and resin in the plant [38,40]. Some studies about the morphological changes of the guayule shrub over time, as well as PI and resin accumulation, try to define the best harvesting time [41].

#### 4.1.1. IPP as Central Precursor Unit

**Figure 6:**
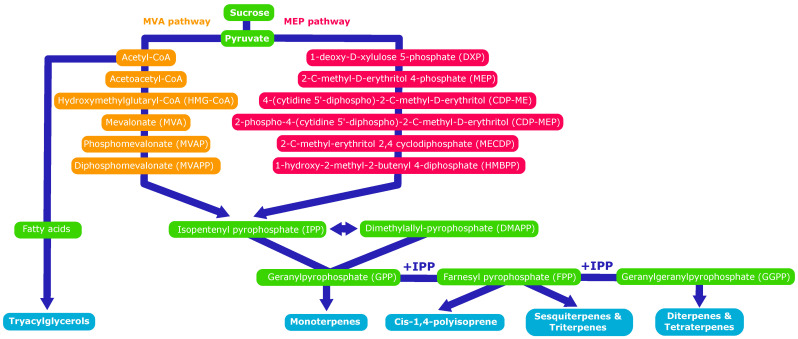
Biosynthesis of polyisoprene and resin compounds.

PI in guayule is *cis*-1,4-polyisoprene, except for the first added monomers which are *trans*-1,4-polyisoprene, in the case of *Hevea* and thus probably also in the case of guayule, based on known similar biosynthetic routes [12,42]. The constituent monomer is isopentenyl pyrophosphate (IPP). It is synthesized from the mevalonate (MVA) pathway or from the 2-C-methyl-D-erythritol-4-phosphate (MEP) pathway [43]. Both syntheses originate from sucrose produced by photosynthesis in the leaves and translocated to the stem [44]. They lead to farnesyl pyrophosphate (FPP), the main basic precursor, formed with dimethylallyl pyrophosphate (DMAPP) and two IPP in *trans*-configuration. Several IPP-units in *cis*-configuration are added to FPP to form the polymer. The two pathways also lead to fatty acids, monoterpenes, sesquiterpenes, diterpenes, triterpenes and tetraterpenes too [45,46]. All of these compounds are synthesized in the epithelial cells and then stored in the resin ducts [47] (Figure 6).

#### 4.1.2. Biosynthesis of Polyisoprene

The biosynthesis of the polymer is catalyzed by a membrane-bound *cis*-prenyl transferase, named rubber transferase (EC 2.5.1.20), part of the monolayer membrane that surrounds cytosolic rubber particles. Polymerization takes place within the membrane monolayer boundary between nonpolar rubber particles and the aqueous medium. It involves three steps: initiation, polymerization, and termination [48].

Guayule PI remains inside the vacuoles, for storage, till plant death. The epithelial cells around resin canals are therefore bifunctional cells: they are first dedicated to the biosynthesis and then they are remodeled to allow PI particles accumulation [38]. PI seems like a “dead-end product” and it was suggested that it acts to prevent damage to the photosynthetic apparatus under conditions of cold temperature, high light or other environmental stimuli [38].

## 5. Chemical Composition of Extractables

### 5.1. Polyisoprene

**Figure 7:**
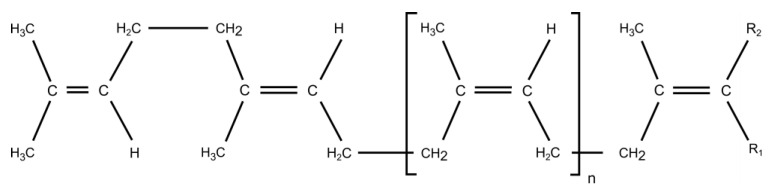
Overview of broad chemical structure of natural polyisoprene (*cis*-1,4 units).

PI in *Hevea* and guayule contains essentially *cis*-1,4 units (Figure 7), unlike the synthetic one. In fact, synthetic polyisoprene results from the polymerization of the 2-methylbuta-1,3-diene. Four polyunsaturated microstructures (1,2; 3,4; *cis*-1,4, *trans*-1,4) are formed, the most interesting configuration being *cis*-1,4. Even if the Ziegler-Natta catalysis allows to reached high percentage of *cis*-1,4 units, natural polyisoprene in NR remains irreplaceable for some applications, like plane and truck tyres [2].

PI with high Mw (1,000,000 g/mol) and with low Mw (<1,000,000 g/mol) is formed in guayule stems, branches and roots [36,49]. A water-based process allows to recover PI chains up to 3,000,000 g/mol [50]. Natural PI chains can be ended by linked phospholipids, fatty acids and their derivatives. They could potentially stiffen the structure [51].

### 5.2. Resin

Resin, contained in all parts of the plant, is a mixture of compounds, generally defined as the acetone extractables. Insoluble wax and higher alcohols are often put apart [52]. Resin contains mostly terpenes and lipids, but also several molecules belonging to other classes of compounds. The following have been reported: monoterpenes (α-pinene, camphene, β-pinene, α-phellandrene, β-phellandrene, sabinene, β-myrcene, limonene, terpinolene, β-ocimene, cadinene, dipentene, bornyl acetate) [52,53,54], sesquiterpenes esters (guayulins A, B, C, D), sesquiterpene alcohols [51,52,53], triterpenes (argentatins A, B, C, D, E, F, G, H) [55,56], alkaloids (guayulamines A, B) [57], organic acids (cinnamic acid) [58], phytosterols [55], triacylglycerols (fatty acids: palmitic, stearic, arachidic, myristic, oleic, linoleic, linolenic) [50,51], flavonoids, flavonoid glycosides [59], and carotenoids [53]. A patent directly tackles the separation of the isoprenic constituents of guayule by liquid-liquid partitioning [60].

**Figure 8:**
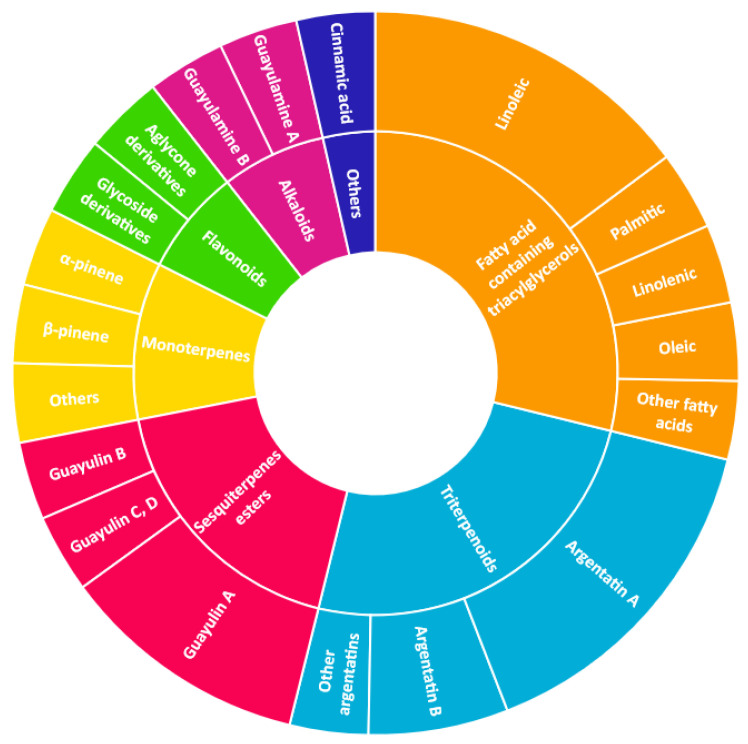
Composition of guayule resin from compiled literature data.

A few studies are focusing on leaves [61,62]. Authors have also extracted an essential oil from the leaves (~2% dry weight) which is mainly a mixture of monoterpenes (α-pinene, 16.7%, camphene, 1.2%, β-pinene, 13.6%, sabinene, 6.5%, β%-myrcene, 2.5%, limonene, 5.9%, terpinolene, 9.2%, β-ocimene, 2.1%), and of sesquiterpenes (39.5%) (Figure 8, Table 2). In the seeds, about 25% of oil (fatty acids: linoleic, 19%, palmitic, 2.5%, stearic, 1.5% oleic, 2%) and 35% of proteins has been found [63,64].

### 5.3. Solid Residues

Guayule bagasse –the solid left after the extraction of PI and resin- is mainly composed of polysacchrarides (holocellulose, 73%; α-cellulose, 43%; pentosane, 16%), lignin, 29%; proteins and oligofructosaccharides (levulins and inulin) [52,65,66]. Estilai et al. [40] and Banigan et al. [52] give the detailed composition of amino acids in the bagasse, which contains also 18% of crude fibers and 1% of sucrose.

## 6. Processes to Extract Dry Rubber and Latex

### 6.1. Industrial Processes

There are two major types of processes for guayule’s PI extraction: processes that extract PI in a coagulated state, the aim being to obtain dry rubber (for tyres for example) and processes that extract PI in the emulsion form (latex for gloves dipping for example).

**Figure 9:**
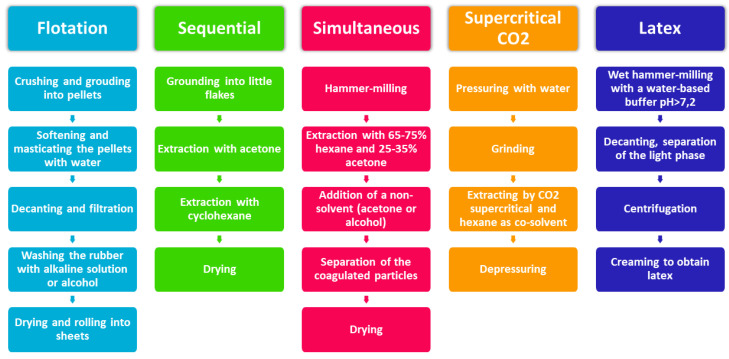
Industrial processes to extract rubber and latex from guayule plants.

**Figure 10:**
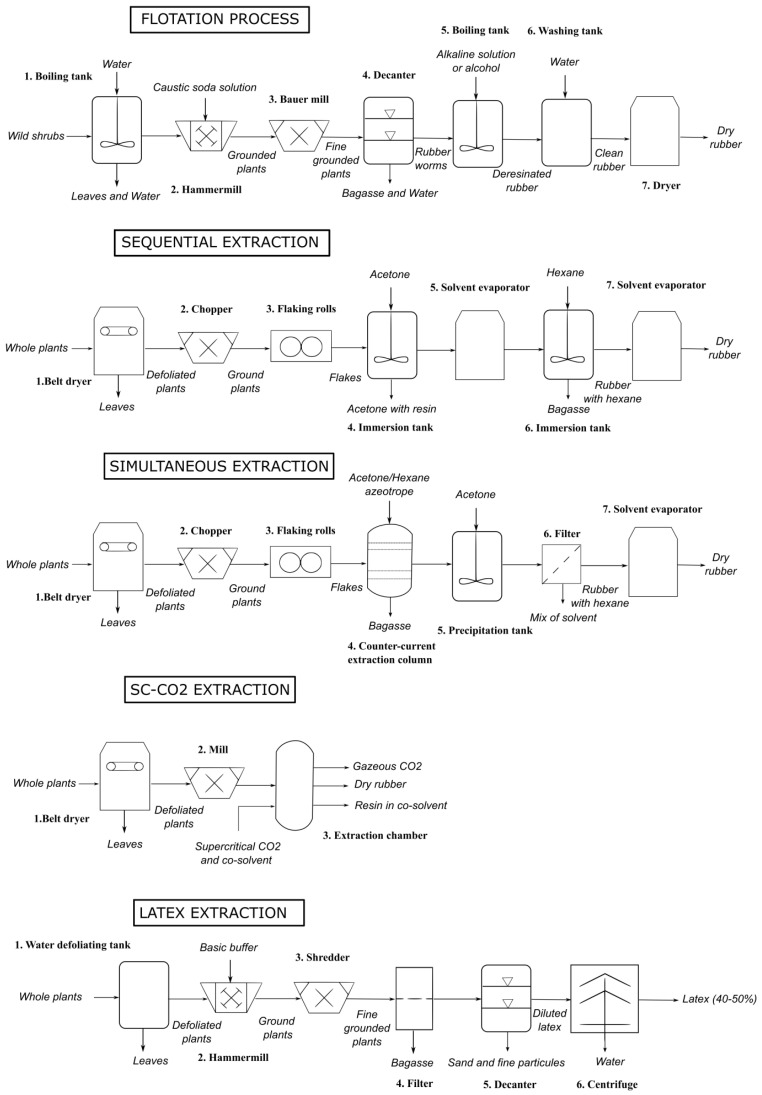
P&ID schemes of proposed industrial processes.

The main processes to obtain dry rubber are: flotation (aqueous process), sequential extraction (solvent process), simultaneous extraction (solvent process), and more recently supercritical-CO_2_ (with co-solvents). To extract PI in the emulsion form, an aqueous process was developed and improved progressively (Figure 9 and Figure 10).

#### 6.1.1. Flotation

The oldest method to extract rubber is flotation. It was the first industrial process used in the early 1900s, in the Mexican pilot plant at Saltillo, Coahuila, operated by the Centro de Investigación en Química Aplicada (CIQA). The wild shrubs are parboiled in hot water, for half an hour. This allows removing leaves and coagulating the rubber in the cells. The plants are then ground in a hammermill and pulped in a Bauer mill, using a water solution with sodium hydroxide to open cells. Milling and softening steps with water are also done in the reverse order. The mixture is then decanted in a tank, until the woody tissue takes-up water, sinks to the bottom, and the resinous rubber floats in what are called “worms”. These worms are skimmed from the top. They usually contain a high proportion of resin. To get rid of the resin, the small masses of gum are treated with an alkaline solution or with alcohol, preferably under high temperature [67]. Rubber is then dried and rolled into rubber sheets. Lawrence and Delafond patented two similar processes with advanced milling techniques [68,69]. They were used by the Intercontinental Rubber Company (ICRC) from 1920 until 1940. Some issues have been raised-up with the process use:

- The treatment with caustic alkali brings a reduction in the acetone soluble content (resin) in the rubber, but the rubber is deteriorated by the long-continued agitation. It loses “nerve”, becoming softer and plastic [70,71].
- The usual method of disposing of this bagasse is to burn it as fuel but, because of the residual rubber, it is an expensive fuel and it implies a great loss to the manufacturer [72].
- Shrubs are harvested and then usually left for a time, which varies greatly, in the sun. This drying negatively affects the quality and the yield of rubber extraction [73,74].
- A lot of rubber is still trapped in the bagasse, but a prolonged grinding in view of increasing the yield reduces the tensile-elongation properties of the rubber and makes it softer and thus more liable to retain fiber and other foreign matter [75].

Some recent discoveries on the final GNR product in the plant helped to create improvements. The resin is adhesive [76], and its presence in the final rubber induces early and rapid degradation during processing and storage [77]. Rubber is present in the form of a colloidal suspension in the plant. It must be completely and correctly coagulated [78]. A lot of improvements were proposed:

- Improvement to clean the raw material: extraction of the rubber with a mixture of acetone, amyl oxyhydrate, methyl oxyhydrate, and alcohol while heating. The resin, oil, and wax can be then separated from solvents by distillation [79]. Another option is to distillate water and essential oil from the obtained rubber [80].
- Improvement of the grinding method: replace the rubs or grinds by a suitable comminuting cut. The main advantage of the process is that it makes possible the use of continuously operating machines.
- Elimination of the resin with a solvent before the original process. The crude woody material can be treated preliminarily with a volatile solvent in which the resin is soluble while the rubber is insoluble (acetone, ethyl alcohol, methyl alcohol), made a much-shortened grinding operation possible, and the particles of rubber display a greater tendency to cohere together than to adhere to other materials [70,81,82].
- Improvement of the separation of the rubber stuck in bagasse: decreasing the specific gravity of the rubber particles, by making them lighter and thus increasing their buoyancy in separating fluid, by adding petroleum-distillate.
- To avoid rubber deterioration, the shrub can be treated with a preservative or stabilizing agent which will stabilize or preserve the rubber: immersion in a tank containing a solution of 1% of dimethyl-paraphenylenediamine [71].
- Improvement of the liberation of the rubber by exposing the shrub to suitable gaseous agent, which penetrates the cells and raises enough pressure to expand instantaneously, like an explosion [74].

This flotation process had quite a few problems, even with the above improvements, so a few different advanced techniques have been developed to overcome such concerns.

#### 6.1.2. Sequential Extraction

Sequential extraction is the oldest solvent process. It has been evaluated in a semi-batch mode at USDA/ARS. After being defoliated, air-dried plants are grounded into little flakes. These are processed through two extraction steps. First, they are extracted with acetone, for removing the resin and then with cyclohexane, for removing GNR. It can be by immersion, gravity percolation or counter current percolation. The solvents are then evaporated. Buchanan patented a sequential solvent process with the action of compressive and shear forces and solvent, being first acetone and then hexane [80]. In this process, a lot of solvent is used and not recycled.

Some improvements have thus been developed:

- Improvement in solvent use: Firestone chose to add a portion of recycled miscella solvent system [83].
- Improvement of pretreatment, chopping and grounding of the plant up to sufficient shear to rupture cell walls and to form agglomerates that can be handled by percolation [84].

#### 6.1.3. Simultaneous Extraction

In the simultaneous extraction process, the entire plant, including leaves, is initially broken down in size into small pieces with a double stage hammer mill. A solvent is added, usually hexane/acetone (75/25), forming an azeotropic mixture. The extraction lasts 1 or 2 h. The rubber and resin are then separated by adjusting the ratio between the polar and nonpolar solvent. Particules are removed by filtration or centrifugation. However, this process presents also some weak points, particularly the fact that solvents are expensive, unless recycled [73].

Some recent improvements have been generated:

- Fractionating rubber by molecular weight using solvents [85].
- Use of different solvents: Bridgestone/Firestone developed a continuous processing using simultaneous extraction with pentane-acetone azeotrope, known as “Simultaneous Extraction and Rubber Fractionation” (SERF) [86].
- Texas A&M University has developed a process based on a batch-mixing screw pressing extraction [87].

#### 6.1.4. Supercritical CO_2_ Process

The plant is first defoliated and then processed into very small pieces. These are contacted with compressed carbon dioxide in the supercritical state with hexane as co-solvent (5000 to 10,000 psi and 60–100 °C for less than an hour) [84].

Cornish has found that the rubber extraction is extremely sensitive to the hexane concentration in the extractor in the course of the extraction cycle. Rubber is only extracted at significant rates when the hexane concentration is extremely high. This team proposed a process using an expanded hexane solvent, rather than a supercritical carbon dioxide one with hexane as co-solvent, with a solvent/feedstock ratio of 2:1 or 3:1 by weight [88]. Worth noting that when using acetone or ethanol as co-solvents under mild supercritical CO_2_ extraction conditions (solvent and CO_2_ weight flow rate ratio 1:15; temperature 33–50 °C; pressure 3000–4500 psi) Punvichai et al. [89] found suitable conditions for selective extraction of PI-free resin components. Therefore, supercritical or pressurized CO_2_ could be used for the cascade extraction of the valuable guayule components by playing with processing conditions in a single extraction tank.

#### 6.1.5. Latex Process

Instead of extracting the PI under dry rubber form, it can be extracted as an emulsion. Spence was the first to propose a latex process [85]. Plants are directly processed upon harvest. They are crushed and milled in a buffer solution (pH > 7.2) to prevent PI coagulation, by maintaining the hydrogen ion concentration close to the plant itself. A diluted latex dispersion is formed, then filtered and put in settling tanks where the fine particles (biomass, sand), are separated. By using centrifugation, a concentrated latex (GNRL; 40% of PI) can be prepared. Eventually a heating and acidifying step allows to obtain coagulated rubber (GNRL) [85]. Like previous processes, some improvements have since been made:

- Improvement of centrifugation: a continuous process using a centrifugal separator to concentrate dilute rubber dispersion in such a way that little or no pasty or firmly coagulated rubber is formed in the bowl to avoid interfering with the efficiency of separation [86].
- Improvement of the cleaning of the product: by cleaning the latex with polar and non-polar solvent (simultaneously or sequentially) or by freezing the latex, or drying the latex to obtain dry rubber [87].
- Improvement of grinding shrub: grinding plants by rotary shearing [50].
- Improvement of latex stabilization: in order to stabilize the latex, grinding of the plants can be made in a buffer containing ammonium hydroxide (or potassium hydroxide, sodium colehydroxide, sodium bicarbonate) and an antioxidant, such as sodium sulfite (or butylated hydroxytoluene, butylated hydroxyanisole) [90].

In all cases authors give useful information, like how to defoliate the shrub [91], to recycle the wastewater [92], to treat the resulting biomass [93] or even how to harvest [94]. The table below summaries the advantages and disadvantages of the different processes.

### 6.2. Analytical Methods

The different industrial processes were adapted for analysis of PI content in the plant and for analysis of PI extractable as latex. Solvent methods are used to assess the PI content in the plant. Researchers choose between an adapted sequential method, accelerated solvent extraction (ASE) (three 20-min cycles at 40 °C with acetone and three 20-min cycles at 120°C with cyclohexane or hexane) [95,96] or a simultaneous extraction, 5 h Soxhlet extraction with pentane: acetone (82:18 *v/v*) [97]. In both cases, the solvent is usually evaporated, and quantification is performed gravimetrically. However Salvucci [96] describes the determination of the resin content by UV absorption directly on the acetone extract, and of the PI content by High Performance Liquid Chromatography (HPLC) coupled to evaporative light scattering (ELS) on the cyclohexane extract, after ASE extraction. Instead of using solvent processes, Suchat and al. [98] and others [99,100,101] proposed Near infrared spectroscopy (NIRS), a non-destructive method, for quick determination of PI and resin content in the plant. This method is based on vibration properties of organic molecules. Chemical bonds can be used to retrieve the chemical composition. The calibration was performed with the ASE method, and attempts are made through measuring directly on the branch, to avoid the biomass grinding step, towards a fast routine technique [102].

To quantify the rubber extractable as latex, Cornish and al. [99] developed a “Waring blender method” and “Oster blender method” in the same vein as the corresponding patent [84].

## 7. Proposed Applications for Guayule-Derived Products

**Figure 11:**
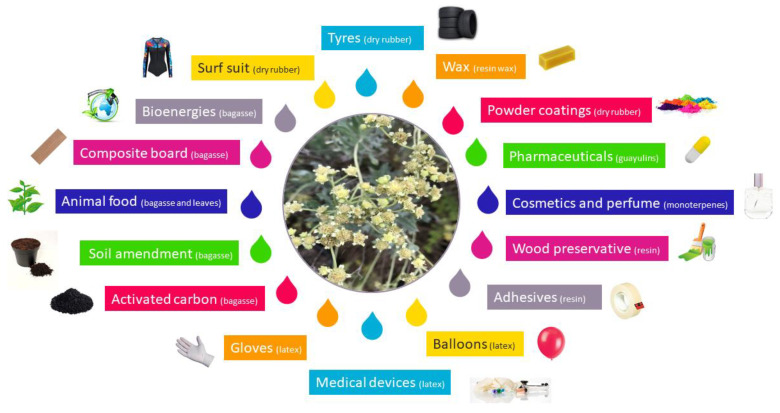
Proposed applications for products and co-products of guayule processing.

Depending on the used process, products and co-products are different: dry rubber GNR or latex GNRL, resin, leaves (if the plant has been defoliated) and bagasse (Figure 11).

### 7.1. Polyisoprene

GNRL could be used for manufacturing numerous medical supplies [103]. Being non-allergenic this latex is a suitable feedstock for surgical tools or examination gloves [4]. These last supplies are made by dipping a form inside tanks containing stabilized and formulated latex [104]. Balloons may also be manufactured with this technique [105].

Tyres and mechanical parts are made with high molecular weight dry rubber, after a vulcanization step [99,101,102]; synthetic neoprene can be replaced by dry natural rubber in surf wetsuit. Low molecular weight guayule rubber has also found applications: rubber hydroxylated by hydroboration/oxidation has been used as powder coatings [106].

### 7.2. Resin

A lot of applications have been proposed for guayule resin. The whole resin can be used as a wood preservative, against damage by *Teredinidae*, and *Limnoria sp.* and particularly, against the very destructive *Coptotermes* termite species [107,108]. Resin shows a prooxidant activity, and it is especially interesting because prooxidants (or peptizers) are ordinarily toxic synthetic products [53]. It can also be used in the formulation of coatings and paints, bringing good properties (abrasion resistance, gloss, drying time, and water resistance) [11,53].

Resin can also be fractionated into different classes of molecules, each having specific properties. Volatiles monoterpenes are similar to those used in the industry of cosmetics and perfume [109].

Argentatins A and B have antimicrobial activities [110] and can also play a role in cancer treatment [111]. Argentatin A is cytotoxic against K562, MCF-7, PC-3, HCT-15 and U251 human cancer cell lines and against proliferating lymphocytes at a concentration of 25 µM, and it also shows a higher potency on prostate cancer cells. It has no cytostatic nor genotoxic effects on lymphocytes at the same concentration. Argentatin B is cytotoxic against the same human cancer lines as argentatin A, but not on proliferating lymphocytes at a concentration of 25 µM. This compound is shown to be better to treat cancer than the currently used ones, which generally induce second malignances [112].

Guayulins A and B have been shown to possess biological properties and to have a key role in the defense of the plant. They actually act as biological triggers in the synthesis of lychnostatine and paclitaxel, which are agents used in breast cancer treatment [113,114].

Wax has similar physicochemical properties as carnauba wax [115], but the total amount of wax is low to be applicable for commercial purposes, unless large quantities become available [52]. Today the main natural source of terpenic resin is the wood and pulp (paper) industry, although having a different chemical composition.

### 7.3. Leaves

Leaf extracts have a high phenolic content and antioxidant activity. They represent a rich source of bioactive compounds, including terpenoids discussed in Section 7.2, interesting for the nutraceuticals and pharmacological areas, but also, simply, as animal feed, being present on the bush all year round [52,116].

### 7.4. Bagasse

The large amount of bagasse resulting from GNR or GNRL extraction process can be directly used for soil amendments. Moreover, by thermochemical conversion, it can also be made into gaseous and liquid fuels [58,117,118], or granular activated carbon [106,116].

## 8. Economic Considerations

Global natural rubber (NR NRL from *Hevea*) production reached to 13.7 million tons in 2018, Asia being the first producer with about 88%, of which 93% was produced by smallholders [119,120]. It is anticipated that the *Hevea* trees cannot support alone the growing demand. Indeed, this crop suffers several constraints affecting production and price stability: a growing demand from the China and India; the South-American leaf blight disease (*Microcyclus ulei*), potentially capable of displacing and destroying *Hevea* plantations in South-East Asia, whereas they are already under attack by another leaf disease due to another fungus (*Pestalotiopsis*). Furthemore, *Hevea* grows in tropical and equatorial areas with high rainfall, and these are not extensible—unless contributing to deforestation—and even shrinking with the development of oil palm and global warming [121,122].

The current market price (June 2020) of *Hevea* rubber (NR) is around 1.12 EUR/kg [123] and that of *Hevea* latex (60%) is less than 1 EUR/kg (around 1.10 USD/kg) [124].

These prices are too low to cover the agronomical and extraction costs. Producing guayule rubber will not be profitable as long as the *Hevea* rubber and latex prices remain at the current level. There is a need to modify the economical equation in order to reach profitability and sustainability.

While state subsidies can change the economical equation, a better sustainable way needs to be found. A look at the companies’ strategies, through their communications, websites, publications, and patents, show several routes [125]:

- Some of them try to reduce production costs by investing in the agronomical practices and extraction process in order to increase the yields. Using varietal selection in order to increase field yield for example.
- Others try to sell manufactured products (like gloves or tyres) rather than raw-materials (latex or rubber) because the last make generally a small part of the end-product price. This is vertical integration.
- Another route is to focus on high-value market, here the latex market rather than the rubber one, for producing non-allergenic latex for high value medical gloves, for example.
- The last way is a kind of horizontal integration by valorizing all coproducts: latex, rubber, resin, bagasse… The bio-refinery path: relying on different products and markets, in order to cover the production cost.

For sure a mixture of all these routes can be even more profitable and sustainable for building this new production chain, guayule products not being marketed today as far as we know.

## 9. Nagoya Protocol

The Nagoya Protocol on Access to Genetic Resources and the Fair and Equitable Sharing of Benefits Arising from their Utilization (ABS) is an international agreement, adopted on October 2010 in Nagoya, Japan [126]. One hundred and twenty three (123) countries have ratified this agreement, including Mexico. The US haven’t up to now. The two main objectives are to protect biodiversity and to share the benefits arising from the use of a country genetic resources. Therefore, it is compulsory to declare the industrial use of guayule seeds to the country of origin. In the present case if the seeds originate from natural stands in the US, no need to declare, but if they are coming from natural stands in Mexico, an agreement with this country has to be edited.

## 10. Future Trends

**Figure 12:**
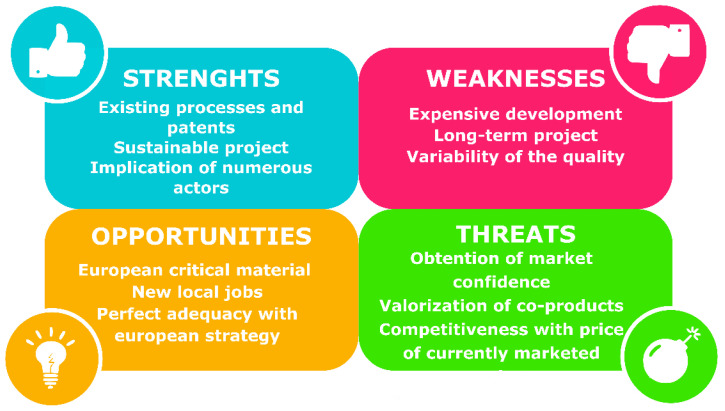
Strengths-Weaknesses-Opportunities–Threats (SWOT) analysis of guayule development.

Guayule is therefore a promising plant for development to complement current production of natural polyisoprene (NR and NRL), and to extract resin, given the wide range of above detailed potential uses. Firms can lean on numerous patents and existing processes, on the implication of numerous stakeholders, in particular in Europe, where natural rubber is on the critical material list of the EU, and in full agreement with its new global sustainable strategy. However, efforts have to be made on co-product valorization to be competitive with currently marketed rubber and latex products (Figure 12).

## Figures and Tables

**Table 1:** Most representative molecules in guayule resin.

Monoterpenes (3–5%) Analysis: GC	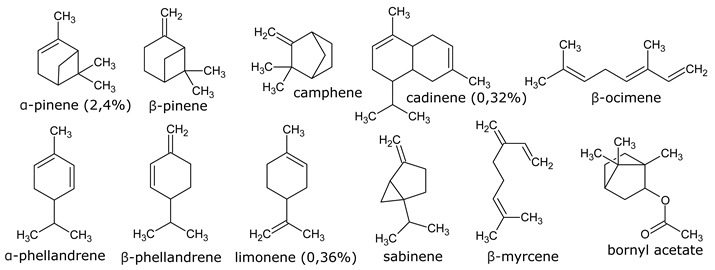
Sesquiterpenes (7–10%) Analysis: HPLC, GC	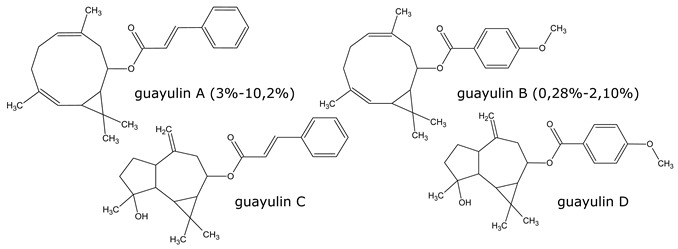
Triterpenes (20–50%) Analysis: HPLC	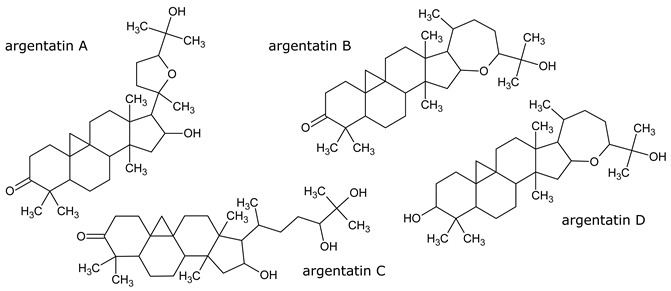
Alkloids Analysis: HPLC, NMR	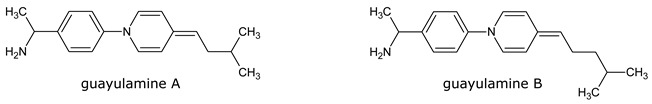
Organic acids Analysis: HPLC	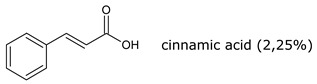
Fatty acid of the triacylglyerols (15–25%) Analysis: GPC, GC	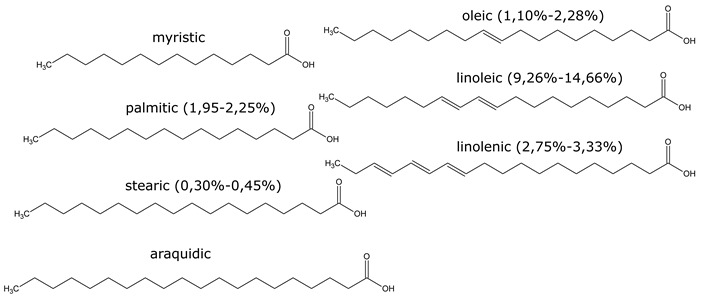

**Table 2:** Summary of the extraction processes.

Process	Advantages	Disadvantages
Flotation Process	Simple, first process Water-based process	Bad quality rubber/lot of resin Important losses
Sequential Extraction	Semi-batch mode Good extraction yield Resin removed by preliminary step	Petrochemical solvents in large quantities
Simultaneous Extraction	One pot extraction Good quality rubber	Petrochemical solvents in large quantities
Supercritical CO_2_ process	Selective extraction of PI One pot extraction Little quantity of co-solvent	Expensive process Special processing conditions
Latex process	PI with high molecular mass Water-based process Continuous process	Difficult to extract in the latex form
